# Preliminary Experience with New Dual-Mobility System for Small Japanese Patients

**DOI:** 10.3390/jcm15093525

**Published:** 2026-05-05

**Authors:** Kenji Kawate, Tomohiro Teranishi, Yumiko Kondo, Mitsumasa Matsui, Shinji Ueno

**Affiliations:** Department of Orthopedic Surgery and Operation Room, Nara Prefecture Seiwa Medical Center, Sango-Town, Nara 636-0802, Japanmatui234679@yahoo.co.jp (M.M.); uensnj1211@gmail.com (S.U.)

**Keywords:** THA, dual mobility, small patient

## Abstract

**Background/Objectives**: The preliminary experience with new dual-mobility system for small Japanese patients was introduced in this paper. **Methods**: Twenty-nine hips which underwent primary THA were retrospectively reviewed. All cups were inserted via Hardinge lateral approach. The ability to perform formal Japanese sitting in a kneeling position (Seiza in Japanese) and bowing while sitting (Zarei in Japanese) was evaluated. The mean follow-up was 6 months. **Results**: The mean age at surgery was 70 years, mean height was 156 cm, mean weight was 58 kg, and mean body mass index was 23.6. The acetabular cups utilized were a hemispherical hydroxy-apatite coated cup (25 hips) and a hemispherical trabecular titanium cup (4 hips), with diameters of 46 mm in 5, 48 mm in 15, 50 mm in 3, 52 mm in 1, 54 mm in 3, 56 mm in 1, and 62 mm in 1; mean diameter was 49.4 mm. No postoperative dislocations including intraprosthetic dislocation or metal allergy were observed. The mean Harris hip score improved significantly from 39 points preoperatively to 89 points postoperatively (*p* < 0.05). Radiographic evaluation demonstrated bone ingrowth stability in all cases according to Engh’s criteria and no aseptic loosening of the implants. Mean hip flexion increased from 75° preoperatively to 90° postoperatively (*p* < 0.05). The ability to perform Seiza increased from 8 patients preoperatively to 23 patients postoperatively (*p* < 0.05). The ability to perform Zarei (deep bowing) increased from 7 patients preoperatively to 20 patients postoperatively (*p* < 0.05). **Conclusions**: This novel dual-mobility system designed for smaller Japanese patients offers three distinct advantages: (1) availability of 42, 44, 46 and 48–66 mm outer diameter cups, (2) 1 mm deeper center of rotation, providing increased jumping distance compared to other designs, and (3) improved assembly instrumentation (cement-gun-type bearing press). Early clinical results suggest that this newly developed dual-mobility THA system is well-suited to the lifestyle and anatomical characteristics of Japanese patients.

## 1. Introduction

Total hip arthroplasty is reported to be the best successful surgical treatment of the 20th century [1]. The indications for revision total hip arthroplasty (THA) include aseptic loosening, infection, and periprosthetic fracture; however, recent data indicate an increasing proportion of recurrent dislocation-related revisions [2,3,4,5]. Causes of dislocation encompass component malposition-induced impingement, bony impingement, capsular and muscular insufficiency, surgical approach (anterior versus posterior), femoral head size (reduced jumping distance), drug or alcohol abuse, prior spinal fusion or fixed spinopelvic alignment and hazardous leg positioning (excessive flexion, adduction, and rotation) [6,7,8,9,10,11,12,13,14,15,16,17,18,19]. Due to traditional lifestyles, deep hip bending activities are often necessary among Japanese people, especially on formal occasions and during religious practices. Developmental dysplasia accounts for 80 percent of the causes of coxarthrosis patients in Japan and small cups (42–48 mm) are often used in THA [20]. The risk of dislocation is relatively high in patients with a small ball and muscle weakness [21,22,23]. Dual-mobility THA, originally developed by Farizon and Bousquet et al. [24] in the 1970s, has demonstrated efficacy in reducing dislocation rates following primary THA and in revision procedures for recurrent dislocation, as reported by many subsequent investigators [25,26,27,28,29,30,31]. Given its proven stability benefits, dual-mobility THA utilization has increased substantially in Japan [32,33,34]. This report describes our initial experience with a novel dual-mobility THA system (Kyocera, Kyoto, Japan) designed specifically for smaller Japanese patients, which has been available since the summer of 2024. This novel dual-mobility system offers three distinct advantages: (1) small-diameter shells, (2) 1 mm deeper center of rotation and (3) improved assembly instrumentation. The purpose of this study was to understand the immediate clinical outcomes of the new dual-mobility system and its adaptability to traditional Japanese lifestyles.

## 2. Materials and Methods

### 2.1. Materials

From July 2024 to October 2025, our department performed THA using the Kyocera dual-mobility system in 29 patients (30 THAs). One male patient who had sustained a periprosthetic fracture in a bicycle traffic accident was excluded. This study comprised 29 consecutive primary THAs. Inclusion criterion: primary THA. Exclusion criteria: patients with a history of metal allergy and patients who had received a traditional polyethylene liner on the opposite side in the past. All patients received a patch test with the same powder material of the metal liner.

The acetabular shells utilized were a hemispherical hydroxy-apatite coated SQRUM AGHA shell and a hemispherical trabecular titanium SQRUM TT shell (KYOCERA Medical, Kyoto, Japan). The metal liner was a cobalt-chromium I-DM liner (mirror-polished inner surface; thickness, 5–6 mm) (KYOCERA Medical, Kyoto, Japan) followed by a vitamin E-infused highly cross-linked polyethylene I-DM bearing (thickness, 5–7 mm) (KYOCERA Medical, Kyoto, Japan). The femoral heads were ceramic Azul balls (alumina—79.3%; zirconia—18.2%; others—2.5%) (KYOCERA Medical, Kyoto, Japan) and a Symarec ball. The femoral stems included a tapered-wedge J-Taper (KYOCERA Medical, Kyoto, Japan), a tapered-wedge INITIA (KYOCERA Medical, Kyoto, Japan), short Optimys (Enovis, Austin, TX, USA), and a 15° de-rotated anatomical short Mainstay (K KYOCERA Medical, Kyoto, Japan).

### 2.2. Surgical Technique

**Figure 1:**
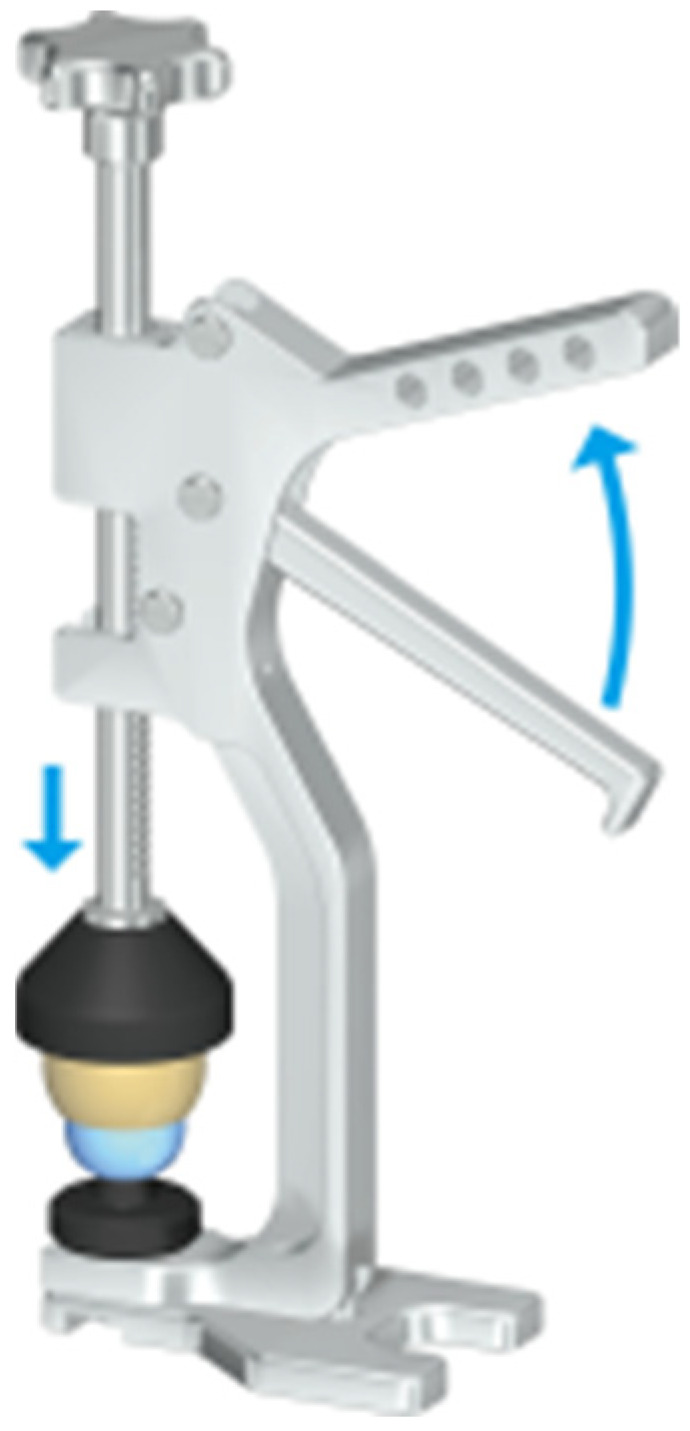
Cement-gun-type bearing press (courtesy of KYOCERA Medical Corp.). The curved arrow showing that the gripping handle act to insert the ball into the polyethylene component.

All procedures were performed via a modified Hardinge lateral approach in the lateral decubitus position. The shell was implanted using the Naviswiss simple navigation system (Naviswiss AG, Brugg, Switserland) with 1 mm under-reaming. The I-DM liner was inserted, followed by assembly of the polyethylene I-DM bearing and femoral head via the bearing press technique (Figure 1). The gluteus minimus and capsule were sutured, and the gluteus medius was also sutured to the original location of the greater trochanter.

The patients were allowed weight bear on the next day and left hospital 2 weeks after surgery. All patients received only standard rehabilitation programs.

### 2.3. Outcomes

All patients received clinical follow-up, during which the Harris hip score was recorded [35]. Antero-posterior (supine position and standing position) and lateral X-rays were taken and evaluated at 3 months, 6 months, 1 year, and 1.5 years after surgery. A final evaluation was performed at the last follow-up. The ability to perform formal Japanese sitting in a kneeling position (Seiza in Japanese) and bowing while sitting (Japanese traditional bowing) (Zarei in Japanese) were also evaluated at the clinical follow-up. Zarei is commonly divided into 4 stages: the first stage is slight bowing (15 degrees), the second is common bowing (30 degrees), the third is salute bowing (45–60 degrees), and the fourth stage is profound bowing (60–90 degrees). Salute and profound bowing are usually considered to be polite in Japan. Therefore, both were judged as Zarei in this study. The bowing angle was measured in the examination room using the same type of protractor at every follow-up.

### 2.4. Statistical Analysis

We performed a paired *t*-test for comparison between the pre- and postoperative Harris hip score, and between pre- and postoperative flexion. Samples were considered as paired, continuous-variable, and normal-distribution data. We also performed the McNemar test for comparison between the pre- and postoperative number of patients who were able to perform Seiza and Zarei. The test statistics were based on a chi-square approximation. Samples were considered as paired, binary-valued, and independent data. Statistical significance was set at *p* < 0.05. Microsoft Excel 365 MSO (Microsoft Corp., Redmond, WA, USA) was used for statistical analysis.

## 3. Results

This study comprised 29 consecutive primary THAs (23 females/25 hips; 4 males/4 hips) with a 3-month minimum follow-up (range, 3–18). The mean age at surgery was 70 years (range, 48–87), mean height was 155.8 cm (144–181), mean weight was 58 kg (35.8–82), and mean BMI was 23.64 (16.2–30.1). Etiologies included developmental dysplasia of the hip (26 hips, Crowe I and II) [36], rapidly destructive coxarthrosis (2 hips), and primary osteoarthritis (1 hip).

The acetabular shells utilized were the SQRUM AGHA shell (25 hips) and SQRUM TT shell (4 hips), with diameters of 46 mm in 5, 48 mm in 15, 50 mm in 3, 52 mm in 1, 54 mm in 3, 56 mm in 1, and 62 mm in 1; the mean diameter was 49.4 mm. The femoral heads were Azul balls in 28 hips and a Symarec ball in 1 hip; 26 mm heads were used with 46 mm shells. The femoral stems included J-Taper (16 hips), INITIA (11 hips), Optimys (1 hip), and Mainstay (1 hip).

Thirteen patients were evaluated at 1 year and 1.5 years after surgery as the last follow-up. No postoperative dislocations, infections, or deep vein thrombosis occurred. No patients complained about pain, clicking, numbness, or nerve paralysis. The Harris hip score improved from a preoperative mean of 39 points (range, 15–67) to a postoperative mean of 89 points (69–100) (*p* < 0.05). Radiographic evaluation demonstrated bone ingrowth stability in all cases according to Engh’s criteria [37] and no aseptic loosening of the implants. Hip flexion improved from a preoperative mean of 75° (20–110°) to 90° (50–120°) (*p* < 0.05). The ability to perform Seiza increased from 8 patients preoperatively to 23 patients postoperatively (*p* < 0.05). Five patients were not able to sufficiently bend at the hips for Seiza, and three patients were unable to bend their knees due to osteoarthritis. The ability to perform Zarei (Stage 3: salute bowing; Stage 4: profound bowing) increased from 7 patients preoperatively to 20 patients postoperatively (Table 1) (*p* < 0.05). Three patients were not able to perform sufficient Zarei because of lower back pain and stiffness. The patients who could perform Zarei also reported ease with standing up from the floor. Most Japanese-style houses have a Tatami mat room; many patients in this study reported that they relax in a Tatami mat room in their homes.

## 4. Discussion

This novel dual-mobility system designed for smaller Japanese patients offers three distinct advantages: (1) availability of 42 (with 22 mm head), 44 (with 22 mm head) and 46 mm (with 26 mm head) outer diameter shells, (2) 1 mm deeper center of rotation, providing increased jumping distance compared to other designs, and (3) improved assembly instrumentation (cement-gun-type bearing press). Small shells are very beneficial to Japanese patients. A total of 80% of Japanese patients suffered from developmental dysplasia of the hip, and most patients were women. The average height of Japanese women over 60 years old is 154 cm, and in those over 70 years old, it is 149.4 cm. In this study, the 46 mm shell was utilized in five hips, expanding treatment options for smaller patients with dysplastic hips. Crowe IV-type hip dysplasia often requires a 42 mm cup [20]. One-millimeter-deeper center of rotation is useful to prevent dislocation. Handling of the cement-gun-type bearing press is easier than that of the vise. A limitation of the cement-gun-type bearing press is that larger polyethylene liners require substantial grip strength for assembly, which proved difficult for us.

Japanese cultural practices including Seiza and Zarei require substantial hip flexion. Satoh et al. [38] reported that more than 60% of routine Japanese living postures had a risk of causing dislocation in THA Patients due to hip joint hyperflexion and adduction, suggesting an increased risk from Japanese living postures. Through four-dimensional motion analysis, Sugano et al. [39] reported that Seiza requires 61 ± 12° and Zarei requires 84 ± 13° of flexion, showing that navigation-guided implant positioning could achieve these ranges without impingement or activity restrictions. They also reported little difference in required hip ROMs between Japanese-style and Western-style activities. All flexion angles with each activity were lower than 120°, which was provided by the combination of cup and stem orientations used in their study for impingement-free ROM. However, many hospitals do not have expensive navigation-guided systems; therefore, it is difficult to obtain an accurate implant position, especially for stem insertion. The movement while sitting on the floor and standing up from the floor is very complicated. Dual-mobility THA expands the margin of error for implant positioning and accommodates complex activities of daily living involving combined flexion, adduction/abduction, and rotation without necessitating activity restrictions. Furthermore, dual-mobility constructs facilitate leg length equalization by eliminating the need for increased leg lengthening to prevent dislocation.

Dual-mobility THA is associated with potential concerns, including destruction of the polyethylene liner from neck-liner impingement, osteolysis, and metallosis from metal ion release, traditionally limiting its use to elderly patients or those with recurrent dislocation [40,41,42,43]. In Japan, osteotomy of the pelvis is recommended for the treatment of dysplastic hip in most patients under 50 years of age. However, the outcome of osteotomy in patients with advanced hip dysplasia is suboptimal. Gamma-eradiated highly cross-linked polyethylene can improve the longevity of THA; therefore, THA is now indicated for younger patients in Japan. Younger patients often need to work for their family and carry out physical labor that requires them to deeply bend their hips. This series included three younger patients (two females aged 48 and 49 years; a male aged 51 years) whose occupations required deep hip flexion; all provided thorough preoperative informed consent. They opted for the dual-mobility THA system because they had jobs as care workers and gardeners, which are roles requiring deep flexion of the hip. Nam et al. reported the use of a dual-mobility prosthesis and a cementless, tapered femoral stem, and showed encouraging results in young, active patients undergoing primary THA [44]. A total of 43 patients (30 male and 13 female; mean age 52.6 years) were included in their study. All patients had a minimum of two years’ clinical follow-up. They reported that mean Harris hip scores improved from 54.1 to 91.2 at two years postoperatively (*p* < 0.001). All patients were evaluated as radiologically well-fixed components, no patients experienced loosening, and no patients required revision surgery. Mean cobalt levels increased at one year postoperatively but decreased at two years. The mean femoral BMD ratio was maintained in Gruen zones 2 to 7 at both one and two years postoperatively. Blakeney et al. [45] reported that ‘modern cups’ with dual mobility have radically changed the situation, with durable implants and therefore potentially new indications for this dual-mobility cup [46]. The current implants have good bone fixation and are durable to loosening [47]. Second, they pointed out the better quality of highly crosslinked polyethylenes. The mechanical properties of the crosslinked polyethylenes would have a good influence on the durability of the dual-mobility implants. Their study encouraged us to use dual-mobility THA for young patients in this study, but the impact of the stem neck on the liner remains anxiety. Further strict follow-up is required for these young patients.

There are several limitations to our study: the small sample size, the short follow-up period, gender bias and the absence of serum metal ion monitoring. Some of these limitations can be attributed to the fact that our institute is located in the countryside, with a low population density. While the patient follow-up was short, the literature suggests that most dislocations occur in the early postoperative period. We wanted to introduce the new dual-mobile cup for small patients as soon as possible in order to make more orthopedic surgeons aware of the existence of the small-sized cup. Additionally, most Japanese patients suffered from dysplastic hips (80%), and they were small female patients; therefore, there was a gender bias in this study. There was also a lack of serum metal ion monitoring in this study; however, some papers reported no increase in serum metal ion at 2 years [48,49], and problematic metallosis was not reported by the longer follow-up studies [50,51]. Long-term follow-up is needed to confirm the durability of this dual-mobility system.

## 5. Conclusions

Although it remains necessary to assess longer-term outcomes, this dual-mobility system appears to be well-suited to the traditional Japanese lifestyle requiring deep flexion activities. The availability of small-outer-diameter shells is useful for smaller patients with dysplastic hips.

## Figures and Tables

**Table 1:** Pre- and postoperative comparison of clinical outcomes.

Parameter	Preoperative	Postoperative	t-Value	*p* Value
Harris Hip Score	39 (15–67)	89 (69–100)	−14.45	*p* < 0.05
Flexion angle	75° (20–110)	90° (50–120)	−3.93	*p* < 0.05
Seiza	8 hips	23 hips	/	*p* < 0.05
Zarei	7 hips	20 hips	/	*p* < 0.05

## Data Availability

The raw data in this study may be obtained from the corresponding author upon reasonable request.

## Abbreviations

THA	Total Hip Arthroplasty
DM	Dual Mobile
I-DM	Initia stem Dual Mobile
